# Study on Gut Microbiota Adaptation of Plateau Zokor (*Eospalax baileyi*) to High-Altitude Environments

**DOI:** 10.3390/microorganisms14071390

**Published:** 2026-06-23

**Authors:** Piao Ma, Fan Ma, Qingfei Hu, Wenjuan Zhang, Haifeng Gu, Dengbang Wei, Zhifang An

**Affiliations:** 1 State Key Laboratory of Plateau Ecology and Agriculture, Qinghai University, Xining 810016, China; 2 School of Ecological and Environmental Engineering, Qinghai University, Xining 810016, China; 3 Qinghai Provincial Key Laboratory of Animal Ecological Genomics, Northwest Institute of Plateau Biology, Chinese Academy of Sciences, Xining 810008, China

**Keywords:** plateau zokor, gut microbiota, metabolites, high-altitude adaptation

## Abstract

To further investigate altitude-associated variations in gut microbiota and serum metabolites of plateau zokors (*Eospalax baileyi*) and elucidate their adaptive mechanisms to high-altitude environments, we performed fecal metagenomic sequencing and serum metabolomic profiling (Q200 platform) on individuals from high (3700 m, *n* = 6) and low (2700 m, *n* = 6) elevations, followed by integrated analysis of microbial and metabolomic datasets. Results indicated that in high-altitude plateau zokors, the relative abundance of Firmicutes decreased, while that of Bacteroidota increased. The dominant genera within this group were identified as *Bacteroides* and unclassified members of the Lachnospiraceae family. Moreover, the abundances of *Bacteroides* and unclassified members of the Muribaculaceae family increased with elevation. At the species level, seven fully annotated differentially abundant taxa were identified: Candidatus *Amulumruptor caecigallinarius*, *Schaedlerella arabinosiphila*, *Muribaculum gordoncarteri*, *Heminiphilus faecis*, *Prevotellamassilia timonensis*, *Staphylococcus aureus*, and *Bacteroides graminisolvens*. KEGG enrichment analysis indicated significant upregulation (*p* < 0.05) of energy supply pathways, such as oxidative phosphorylation, and antioxidant-related pathways, including β-alanine and lysine metabolism, in the high-altitude group. Conversely, cysteine and methionine metabolism pathways were markedly downregulated (*p* < 0.05). Serum levels of ursodeoxycholic acid and tauroursodeoxycholic acid (TUDCA) were significantly elevated (*p* < 0.05), while deoxycholic acid (DCA) levels decreased (*p* < 0.05). In conclusion, the composition and function of gut microbiota, along with serum metabolite profiles, differ significantly (*p* < 0.05) between plateau zokors from different altitudes. Through synergistic interactions between gut microbiota and host metabolites, plateau zokors develop adaptive mechanisms that integrate energy metabolism, oxidative stress response, intestinal barrier integrity, and mucosal immunity. This ultimately facilitates their acclimatization to high-altitude extreme environments characterized by hypoxia and low temperatures.

## 1. Introduction

The gut microbiota is integral to host physiology, performing critical functions such as food digestion, energy harvest, and immune regulation [1,2,3]. Its composition is governed by the dynamic interplay and co-evolution between the host and its environment [4]. The Qinghai–Tibet Plateau presents a unique set of environmental challenges, including low-temperature conditions, intense solar radiation, reduced atmospheric pressure, and hypoxic conditions; nevertheless, it supports considerable biodiversity. Through long-term adaptive evolution, indigenous alpine animals on the plateau have developed a range of survival strategies. The gut microbiota of these animals further contributes to host adaptation to such extreme conditions through metabolic and immunomodulatory mechanisms and is implicated in shaping behavioral phenotypes [5]. In high-altitude environments, the host gut microbiota exhibits an increased abundance of Bacteroidetes and Verrucomicrobia alongside a decreased abundance of *Firmicutes* [6,7]. This altered community profile enhances carbohydrate metabolism and short-chain fatty acid (SCFA) production, thereby helping the host meet energy demands in hypoxic and low-temperature environments [6,7]. The high-altitude environment significantly influences the gut microbiota of plateau animals, and the microbial composition of endemic plateau animal species exhibits distinct species specificity [8,9,10]. Gut microbiota not only facilitate nutrient and energy acquisition for the host but also produce various metabolites that play pivotal roles in regulating both the innate and adaptive immune systems [11]. Studies have shown that reduced levels of gut microbial metabolites, such as SCFAs, bile acids, and their derivatives, may disrupt the signaling crosstalk between gut microbiota and intestinal immune function, contributing to disease development [12].

The plateau zokor (*Eospalax baileyi*), a small mammal endemic to the Qinghai–Tibet Plateau, is a member of the genus *Eospalax* within the family Spalacidae (order Rodentia) [13,14,15]. This species inhabits sealed burrows throughout its lifetime and is widely distributed throughout the eastern Qinghai-Tibet Plateau at elevations of 2800–4200 m [16]. High-altitude environments—characterized by extreme cold, aridity, intense ultraviolet radiation, and hypoxia—pose substantial survival challenges for mammals [17]. Through long-term evolution, the plateau zokor has developed numerous physiological and molecular adaptations to survive hypoxic conditions [18,19,20]. While most current research focuses on the species composition of gut microbiota, studies investigating the high-altitude adaptation mechanisms of plateau zokors from the perspectives of functional gene profiles and microbial metabolites remain limited. To investigate altitudinal variations in the gut microbiota and metabolites of plateau zokors, this study employed metagenomic sequencing of fecal samples to analyze microbial composition and functional diversity, alongside Q200 metabolomic sequencing of serum to profile metabolites. Correlation analyses between differential microbial taxa and metabolites were performed to elucidate their interactions. This approach enhances our understanding of the gut microbial community structure and metabolic profile of plateau zokors, providing insights into how wild rodents adapt to extreme high-altitude environments.

## 2. Materials and Methods

### 2.1. Laboratory Animals

Plateau zokors from the high-altitude group (ZH) were captured at the Laji Mountain (36°21′ N, 101°25′ E; 3700 m above sea level) in Guide County, Hainan Tibetan Autonomous Prefecture, Qinghai Province (Qinghai, China). At this site, the atmospheric pressure was 59 kPa, with a partial pressure of oxygen of 11.74 kPa and an oxygen content of 173.1 g/m^3^. Zokors from the low-altitude group (ZL) were captured in Shangquan Village (36°19′ N, 101°38′ E; 2700 m above sea level), Huangzhong County, Xining City, Qinghai Province (Qinghai, China). This location exhibited an atmospheric pressure of 70 kPa, a partial pressure of oxygen of 14.69 kPa, and an oxygen content of 197.6 g/m^3^. Sampling was conducted in September 2023, with six individuals per group. The animals were anesthetized via intraperitoneal injection of 20% urethane (Solarbio, Beijing, China) (1 g/kg body weight), and blood was collected from the carotid artery. Following blood collection, fecal samples from the colon were immediately gathered and snap-frozen in liquid nitrogen. The blood samples were allowed to clot at 4 °C for 30 min, centrifuged at 3000 rpm for 10 min to obtain serum, which was then aliquoted and stored in liquid nitrogen.

All experimental protocols involving animals were approved by the Science and Technology Ethics Committee of Qinghai University and were performed in strict compliance with China’s national standard Laboratory Animal—Guideline for Ethical Review of Animal Welfare (GB/T 35892-2018) [21]. The disposal of animal carcasses was recorded and managed by the Animal Experiment Management Office of the State Key Laboratory at Qinghai University.

### 2.2. Metagenomic Sequencing

Genomic DNA was extracted from the samples using the Mag-Bind® Soil DNA Kit (Omega Bio-Tek, Norcross, GA, USA). The concentration and purity of the DNA were quantified, and its integrity was verified through 1% agarose gel electrophoresis. The DNA was sheared to an average fragment size of approximately 350 bp using a Covaris M220 focused ultrasonicator (Covaris, Woburn, MA, USA). Following size selection for fragments of approximately 350 bp, paired-end (PE) libraries were prepared using the NEXTFLEX™ Rapid DNA-Seq Kit (Bioo Scientific, Austin, TX, USA) and sequenced on an Illumina NovaSeq 6000 platform (Illumina, San Diego, CA, USA).

Raw sequencing reads underwent processing that included demultiplexing, adapter trimming, quality filtering, and removal of contaminant sequences. The resulting high-quality reads were de novo assembled, and protein-coding genes were predicted from the assembled contigs. These predicted genes were annotated against taxonomic and functional databases, yielding data on taxonomic composition, functional profiles, relative abundance, and differential abundance. Raw sequencing reads were subjected to quality control using Fastp v0.20.0 to obtain high-quality sequences. The sequences were then assembled into contigs using MEGAHIT v1.2.9. Open reading frames (ORFs) were predicted with Prodigal v2.6.3, excluding sequences shorter than 100 bp. All predicted ORFs were subsequently clustered with CD-HIT to construct a non-redundant gene set. For taxonomic annotation, gene sequences were aligned to the NCBI NR database using Diamond v2.0.13. Additionally, functional annotation was performed against the KEGG, COG, CAZy, CARD, ARDB, and VFDB databases.

### 2.3. Q200-Based Untargeted Metabolome Sequencing

Fecal and serum metabolites from plateau zokors were quantified using Q200 macro-metabolomics technology. The Q200 platform enables targeted quantification of over 200 host-microbiota co-metabolites spanning major classes such as indoles, carbohydrates, bile acids, fatty acids (including short-chain fatty acids), organic acids, acylcarnitines, amino acids, phenylpropionic acids, and phenyl-acyl compounds. For serum analysis, 20 µL of each sample was transferred into a 96-well plate using an Eppendorf epMotion automated liquid handling workstation (Eppendorf Inc., Hamburg, Germany). Ice-cold methanol (120 µL) containing internal standards was then added to each well. The plate was vortex-mixed vigorously for 5 min and centrifuged. Subsequently, 20 µL of freshly prepared derivatization reagent was added to each well. The plate was sealed and incubated at 30 °C for 60 min to allow derivatization to proceed to completion. In parallel, a calibration series was prepared by serially diluting derivatized standard stock solutions. Finally, the plate was re-sealed and analyzed using an ACQUITY UPLC-Xevo TQ-S triple quadrupole mass spectrometer coupled to an ultra-performance liquid chromatography system (Waters Corp., Milford, MA, USA). Metabolite quantification was performed via tandem mass spectrometry (MS/MS) in multiple reaction monitoring (MRM) mode.

### 2.4. Integrated Analysis of Differential Gut Microbiota and Serum Metabolites

To investigate the relationship between gut microbiota and their metabolites in plateau zokors, we first extracted abundance data for differentially abundant gut microbes and metabolites from the original omics datasets. Subsequently, we employed Spearman’s correlation analysis [22] to conduct cross-omics association analysis between the relative abundance of differential microbial taxa and corresponding metabolites, thereby evaluating correlations between gut microbial communities and their associated metabolites.

### 2.5. Statistical Analysis

In this metagenomic analysis, we assessed overall changes in the gut microbial community structure of plateau zokors using alpha diversity metrics and beta diversity metrics (based on the Jaccard distance). Differential microbial taxa were identified using Linear Discriminant Analysis Effect Size (LEfSe), and differential metabolic pathways were further identified via Kyoto Encyclopedia of Genes and Genomes (KEGG) enrichment analysis. For targeted metabolomics, orthogonal partial least-squares discriminant analysis (OPLS-DA) was applied to evaluate the stability and reliability of serum metabolic profiles. Differential metabolites were screened according to variable importance in the projection (VIP) values from multivariate analysis, combined with univariate *p*-values and fold-change (FC) values. Differences in the relative proportions of functional pathways between the high-altitude and low-altitude groups were assessed using the Wilcoxon rank-sum test. Finally, Spearman’s correlation coefficients were calculated to explore associations between differential microbial taxa and metabolites. All statistical graphs were generated using GraphPad Prism 10, with results expressed as mean ± standard deviation. Statistical significance was defined as *p* < 0.05.

## 3. Results

### 3.1. Metagenomic Analysis of Plateau Zokor Colonic Contents

#### 3.1.1. Analysis of Gut Microbial Diversity in Plateau Zokor

**Figure 1:**
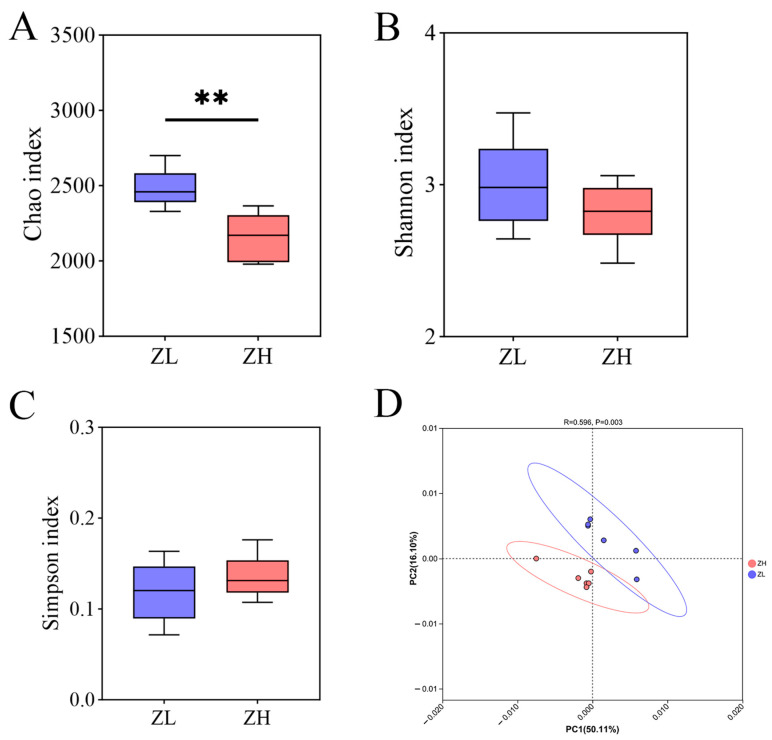
Gut microbial alpha diversity and beta diversity in plateau zokors. (**A**) Chao index; (**B**) Shannon index; (**C**) Simpson index; (**D**) Principal coordinate analysis (PCoA). (ZL) High-altitude plateau zokors; (ZH) Low-altitude plateau zokors. Significance levels: ** *p* < 0.01.

The alpha diversity results for the gut microbiota are illustrated in Figure 1A–C. A significant decrease in the Chao index was observed with increasing altitude (*p* < 0.05), indicating a reduction in microbial richness within the high-altitude group. To visualize the differences in microbial community composition among the groups, Principal Coordinates Analysis (PCoA) was employed. Figure 1D demonstrates a clear separation between the high-altitude and low-altitude groups, suggesting distinct compositions of gut microbiota.

#### 3.1.2. Composition and Differential Analysis of Gut Microbiota in Plateau Zokors

**Figure 2:**
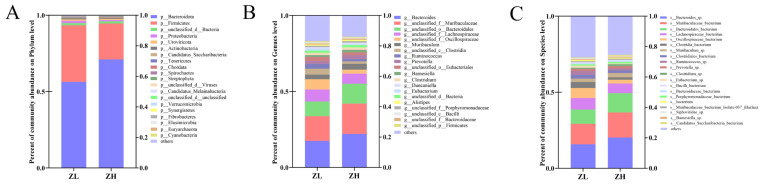
Analysis of composition and abundance of gut microbiota in plateau zokors. (**A**–**C**) represent the microbial community composition at the phylum, genus, and species levels, respectively. (ZL) High-altitude plateau zokors; (ZH) Low-altitude plateau zokors.

At the phylum level, the ten most abundant microbial taxa included Bacteroidota, Firmicutes, unclassified_d__Bacteria, Proteobacteria, Uroviricota, Actinobacteriota, candidatus_d__saccharibacteria, Tenericutes, Chordata, and Spirochaetes. Among these, Bacteroidota and Firmicutes emerged as the most abundant and dominant phyla. Notably, the high-altitude group showed higher Bacteroidota abundance and lower Firmicutes abundance than the low-altitude group (Figure 2A).

At the genus level, the ten most abundant taxa in the gut microbiota of plateau zokor were *Bacteroides*, unclassified_f__Muribaculaceae, unclassified_o__Bacteroidales, unclassified_f__Lachnospiraceae, unclassified_f__Oscillospiraceae, *Muribaculum*, unclassified_ c__Clostridia, *Ruminococcus*, and *Prevotella*. The dominant genera identified were *Bacteroides* and unclassified_f__Lachnospiraceae. In comparison to the low-altitude group, the high-altitude group exhibited increased abundances of *Bacteroides* and unclassified_f__Muribaculaceae, while the abundances of *Muribaculum* and unclassified_f__Oscillospiraceae were reduced (Figure 2B).

Based on metagenomic sequencing, species-level analysis revealed that the ten most abundant OTUs were annotated solely as unclassified strains or unidentified species at the family or genus level, without clear species identification.

Based on metagenomic sequencing, species-level analysis revealed that the ten most abundant operational taxonomic units (OTUs) were annotated solely as unclassified strains or unidentified species at the family or genus level, without clear species identification. These taxa predominantly included *Bacteroides*, Muribaculaceae, and Lachnospiraceae (Figure 2C). Overall, the trends in species-level abundance distribution were consistent with those observed at the genus level.

#### 3.1.3. Screening and Identification of Differential Gut Microbes in Plateau Zokors at Different Altitudes

**Figure 3:**
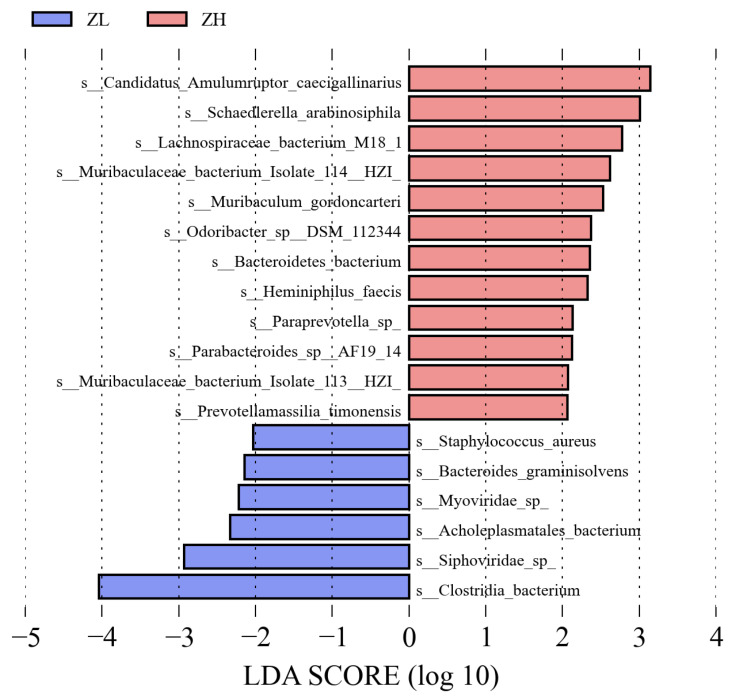
LEfSe differential analysis of gut microbiota in plateau zokors. (ZL) High-altitude plateau zokors; (ZH) Low-altitude plateau zokors.

To further identify gut microbial taxa that significantly differed between the high- and low-altitude groups, we applied LEfSe analysis at the species level to metagenomic sequencing data (LDA > 2, *p* < 0.05), resulting in the identification of 18 differentially abundant taxa (Figure 3). Based on the completeness of their taxonomic annotation, these taxa were categorized into three groups. The first group, which was fully annotated to the species level, included Candidatus *Amulumruptor caecigallinarius*, *Schaedlerella arabinosiphila*, *Muribaculum gordoncarteri*, *Heminiphilus faecis*, *Prevotellamassilia timonensis*, *Staphylococcus aureus*, and *Bacteroides graminisolvens*. The second group lacked species-level resolution and could only be reliably classified at the genus level, comprising *Odoribacter* sp. DSM 112344, *Paraprevotella* sp., and *Parabacteroides* sp. AF19_14. The third group had insufficient taxonomic information and could only be assigned to higher ranks (e.g., phylum, class, or family) and included Lachnospiraceae bacterium M18_1, Muribaculaceae bacterium Isolate I114_HZI, Muribaculaceae bacterium Isolate I113_HZI, Bacteroidetes bacterium, Myoviridae sp., Siphoviridae sp., Acholeplasmatales bacterium, and Clostridia bacterium.

Among the differentially abundant taxa enriched in the high-altitude group, five taxa were identifiable to the species level: Candidatus *Amulumruptor caecigallinarius*, *Schaedlerella arabinosiphila*, *Muribaculum gordoncarteri*, *Heminiphilus faecis*, and *Prevotellamassilia timonensis*. In contrast, three taxa were resolvable only to the genus level: *Odoribacter* sp. DSM 112344, *Paraprevotella* sp., and *Parabacteroides* sp. AF19_14. In the low-altitude group (ZL), the taxa identifiable to the species level included *Staphylococcus aureus* and *Bacteroides graminisolvens*. Collectively, these ten taxa, with complete taxonomic annotation and unambiguous classification, serve as key microbial biomarkers for subsequent multi-indicator analyses.

#### 3.1.4. KEGG Functional Analysis of Gut Microbiota in Plateau Zokors

**Figure 4:**
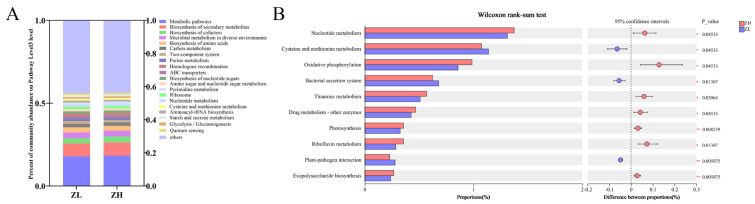
Functional analysis of gut microbiota in plateau zokors. (**A**) KEGG functional enrichment in plateau zokors; (**B**) Differentially abundant KEGG functions in plateau zokors. (ZL) High-altitude plateau zokors; (ZH) Low-altitude plateau zokors. Significance levels: * *p* < 0.05, ** *p* < 0.01.

KEGG pathway enrichment analysis at level 3 revealed that the most significantly enriched pathways in the gut microbiota of plateau zokors included metabolic pathways, biosynthesis of secondary metabolites, biosynthesis of cofactors, microbial metabolism in diverse environments, and biosynthesis of amino acids (Figure 4A).

We further conducted a differential analysis of gut microbial functions between high- and low-altitude plateau zokors. Statistical analysis was performed on the top 20 differential pathways. Compared to the low-altitude group, high-altitude zokors exhibited significant upregulation in pathways such as nucleotide metabolism, oxidative phosphorylation, thiamine metabolism, drug metabolism (other enzymes), photosynthesis, riboflavin metabolism, exopolysaccharide biosynthesis, valine, leucine, and isoleucine degradation, beta-alanine metabolism, lysine degradation, and glucosinolate biosynthesis. Conversely, pathways including cysteine and methionine metabolism, bacterial secretion system, plant-pathogen interaction, microRNAs in cancer, longevity-regulating pathway (worm), pathways in cancer, PI3K-Akt signaling pathway, antigen processing and presentation, and chemical carcinogenesis (receptor activation) were significantly downregulated in the high-altitude group (Figure 4B).

### 3.2. Serum Metabolite Analysis of Plateau Zokors

**Figure 5:**
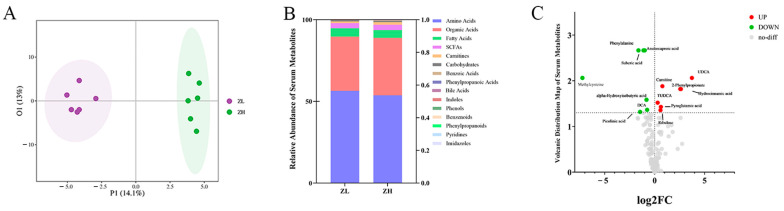
Metabolite analysis of plateau zokors. (**A**) OPLS-DA analysis of serum metabolites in plateau zokors; (**B**) Abundance profiles of serum metabolites in plateau zokors; (**C**) Volcano plot of serum metabolites in plateau zokors. (ZL) High-altitude plateau zokors; (ZH) Low-altitude plateau zokors.

To assess the differences in serum metabolite composition between high- and low-altitude plateau zokors, principal component analysis (PCA) and orthogonal partial least squares-discriminant analysis (OPLS-DA) were performed on all samples. As shown in Figure 5A, the serum metabolites from the high-altitude and low-altitude groups formed two distinct clusters, demonstrating high intra-group consistency and significant inter-group differences.

Targeted metabolomics was conducted, and the relative abundances of metabolite categories were visualized in a stacked bar chart (Figure 5B). The most abundant categories included amino acids, organic acids, fatty acids, short-chain fatty acids, and carnitine. Compared to the low-altitude group, levels of amino acids and carnitine decreased in the high-altitude group, while levels of organic acids and fatty acids increased.

Differential metabolites were identified based on specific criteria: Student’s t-test from univariate analysis (*p* < 0.05), fold-change values (log_2_FC ≥ 0), and variable importance in projection (VIP > 1) as determined by the orthogonal partial least-squares discriminant analysis (OPLS-DA) multivariate model. As a result, 15 significantly differential metabolites were identified in the serum of plateau zokors between the high-altitude and low-altitude groups. To emphasize these key differential metabolites, a volcano plot was generated based on the original data (Figure 5C). The metabolites significantly upregulated in the high-altitude group included carnitine, hydrocinnamic acid, pyroglutamic acid, ribulose, TUDCA, ursodeoxycholic acid (UDCA), and 2-phenylpropionic acid. In contrast, the metabolites significantly downregulated were aminocaproic acid, phenylalanine, methylcysteine, DCA, suberic acid, picolinic acid, and α-hydroxyisobutyric acid.

The classification of differential metabolites revealed specific trends across various categories. In the amino acid metabolite category, levels of phenylalanine and methylcysteine were significantly decreased, while pyroglutamic acid exhibited a significant increase in the high-altitude group. Regarding carbohydrate metabolites, ribulose was notably upregulated at high altitude. Among fatty acid metabolites, suberic acid was significantly reduced in the high-altitude group. In the phenylpropanoid metabolite category, both 2-phenylpropionic acid and hydrocinnamic acid showed significant increases. For bile acid metabolites, UDCA and TUDCA were significantly upregulated, whereas DCA in the high-altitude group was significantly downregulated. In the carnitine metabolite category, carnitine itself was significantly increased. Within pyridine metabolites, picolinic acid was significantly decreased in the high-altitude group. Lastly, among organic acid metabolites, aminocaproic acid and α-hydroxyisobutyric acid were significantly reduced in the high-altitude group.

### 3.3. Correlation Analysis of Differential Gut Microbiota and Serum Metabolites in Plateau Zokors

**Figure 6:**
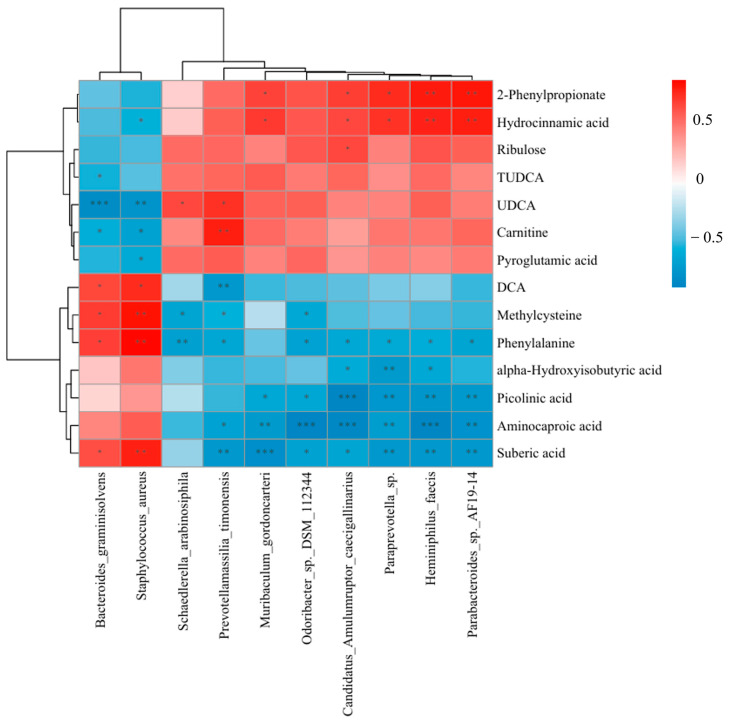
Combined analysis of differential gut microbiota and serum differential metabolites in plateau zokors. Significance levels: * *p* < 0.05, ** *p* < 0.01, *** *p* < 0.001.

Spearman’s rank was used to evaluate associations between differentially abundant gut microbes, identified through metagenomics, and differential serum metabolites, screened via metabolomics, in plateau zokors (Figure 6). Notably, *Bacteroides graminisolvens* and *Staphylococcus aureus* exhibited similar correlation patterns: both demonstrated significant positive correlations with DCA, methylcysteine, phenylalanine, and suberic acid, while showing negative correlations with carnitine and UDCA. Furthermore, *Staphylococcus aureus* was significantly negatively correlated with hydrocinnamic acid and pyroglutamic acid. *Schaedlerella arabinosiphila* was significantly positively correlated with UDCA and negatively correlated with methylcysteine and phenylalanine. *Muribaculum gordoncarteri* displayed significant positive correlations with 2-phenylpropionate and hydrocinnamic acid, and negative correlations with picolinic acid, aminocaproic acid, and suberic acid. Candidatus *Amulumruptor caecigallinarius*, *Paraprevotella* sp. and *Heminiphilus faecis* exhibited consistent association patterns, all three were significantly positively correlated with 2-phenylpropionate and hydrocinnamic acid, while showing significantly negative correlations with phenylalanine, α-hydroxyisobutyric acid, picolinic acid, aminocaproic acid, and suberic acid. *Parabacteroides* sp. AF19-14 exhibited a significant positive correlation with 2-phenylpropionate and hydrocinnamic acid, while showing a negative correlation with phenylalanine, picolinic acid, aminocaproic acid, and suberic acid. In contrast, *Odoribacter* sp. DSM 112344 did not demonstrate any significant positive correlations; however, it was significantly negatively correlated with methylcysteine, phenylalanine, picolinic acid, aminocaproic acid, and suberic acid. Additionally, *Prevotellamassilia timonensis* showed a significant positive correlation with carnitine and UDCA, alongside negative correlations with DCA, methylcysteine, phenylalanine, aminocaproic acid, and suberic acid. These findings suggest that the differentially abundant microbes are intricately linked to core metabolites, including amino acids and bile acids, indicating a potential role in influencing host adaptation to high-altitude hypoxia by modulating amino acid and bile acid metabolic pathways through the gut microbiota.

**Figure 7:**
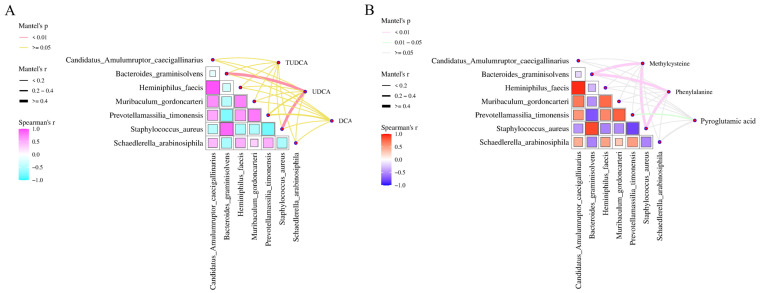
Correlation network heatmap analysis between differential gut microbiota and serum differential metabolites in plateau zokors. (**A**) Differential microorganisms and differential bile acids; (**B**) Differential microorganisms and differential amino acids.

To explore the interactions and regulatory patterns between gut microbiota and host circulating metabolites in high- and low-altitude habitats, we selected seven named differential gut microorganisms: Candidatus *Amulumruptor caecigallinarius*, *Schaedlerella arabinosiphila*, *Muribaculum gordoncarteri*, *Heminiphilus faecis*, *Prevotellamassilia timonensis*, *Staphylococcus aureus*, and *Bacteroides graminisolvens*. We performed correlation network heatmap analysis with significantly differentiated amino acids (pyroglutamic acid, cysteine methyl ester, phenylalanine) and bile acids (UDCA, TUDCA, DCA). In Figure 7, the left heatmap presents Spearman correlations among the differential microorganisms, where the color depth and area size of the squares indicate the sign and magnitude of the correlation coefficients, respectively. The connections on the right depict Mantel correlations between gut microorganisms and corresponding metabolites, with line color and thickness reflecting the significance level and correlation strength. The results indicated that *Bacteroides graminisolvens* and *Staphylococcus aureus* exhibited highly congruent abundance trends, both displaying significantly positive correlations with ursodeoxycholic acid (UDCA), cysteine methyl ester, and phenylalanine. Additionally, *Prevotellamassilia timonensis* was significantly correlated with pyroglutamic acid.

## 4. Discussion

The gut microbiota plays a crucial role in the adaptation of animals to extreme environments [23]. Long-term resident mammals of the Qinghai–Tibet Plateau have evolved integrated physiological and molecular regulatory systems to withstand hypoxic, frigid extreme habitats [18,19,20]. As a significant environmental stressor, high altitude profoundly influences the structure and composition of the host gut microbiota [23]. Previous studies have shown that the gut microbiota of various plateau animals, including Tibetan sheep [24], plateau pikas [25], Tibetan antelopes and kiangs [8], undergoes adaptive reshaping with increasing altitude. Moreover, cold stress, another critical environmental factor on the plateau, significantly alters the α-diversity and community composition of the mammalian gut microbiota [26,27,28]. The plateau zokor, which permanently inhabits extreme underground environments characterized by high altitude, hypoxia, and low temperature, also exhibits adaptive changes in its gut microbiota in response to these environmental pressures [29]. This study demonstrates that the Chao index of the gut microbiota in plateau zokors significantly declines with increasing altitude, indicating a reduction in microbial richness under high-altitude conditions. Furthermore, principal coordinate analysis (PCoA) reveals a pronounced divergence in gut microbial community structure between high- and low-altitude populations. These findings confirm that high-altitude stress significantly shapes the gut microbiota composition of plateau zokors, consistent with observations in other high-altitude mammals, where gut microbiota undergo adaptive changes along elevational gradients. Therefore, plateau-dwelling small mammals may adapt to extreme environments through adjustments in gut microbiota composition and structure.

Long-term exposure to high-altitude environments induces coordinated adaptive changes in the gut microbiota of plateau animals across multiple taxonomic levels (e.g., phylum, genus, and species). At the phylum level, as altitude increases, the relative abundance of Firmicutes in tree sparrows significantly increases, while that of Proteobacteria decreases, a shift further modulated by seasons [23]. In cold-season Tibetan sheep, Bacteroidetes are found to increase while Firmicutes decrease [24], with similar shifts observed in plateau pikas [25]. Plateau pikas also demonstrate an increase in Bacteroidetes and a decrease in Firmicutes [30], aligning with our findings. In the present study, Firmicutes and Bacteroidetes dominate the gut microbiota of plateau zokors, with Bacteroidetes increasing and Firmicutes decreasing at higher altitudes. Bacteroidetes are known to decompose complex carbohydrates to provide energy for the host [31], exhibit strong polysaccharide metabolic capabilities, and are closely associated with metabolic regulation and immune defense [32]. Firmicutes, on the other hand, ferment fiber to produce short-chain fatty acids, which play a crucial role in energy acquisition [33]. These findings suggest that as altitude increases, plateau animals upregulate Bacteroidetes to enhance the decomposition of complex carbohydrates and energy supply, while moderately downregulating Firmicutes to optimize energy acquisition efficiency.

At the genus level, *Ruminococcus*, *Oscillospira*, and *Clostridium* are common core taxa among high-altitude herbivores, including Tibetan antelope, Tibetan wild ass, and Tibetan sheep. Ruminococcaceae and *Clostridium* are known to produce short-chain fatty acids, thereby supplying energy to the host [8]. In Tibetan pigs, the abundances of *Pseudoflavonifractor*, *Parabacteroides*, and *Butyrivibrio* are elevated, collectively promoting the production of short-chain fatty acids, which are critical for intestinal health and metabolic function [34]. Our study revealed that the gut microbiota of plateau zokors harbored high abundances of genera including *Bacteroides*, *Ruminococcus*, and *Prevotella*. *Prevotella* plays a key role in modulating the peroxisome proliferator-activated receptor signaling pathway [35] and exhibits a positive correlation with short-chain fatty acid-specific receptors [36]. These findings indicate that, at the genus level, plateau zokors maintain intestinal health and immune homeostasis in high-altitude environments by harboring high abundances of genera such as *Bacteroides* and *Ruminococcus*.

At the species level, we identified several microbial taxa that significantly differed between the gut microbiota of plateau zokors from high- and low-altitude environments. These include *Schaedlerella arabinosiphila*, *Muribaculum gordoncarteri*, *Bacteroides graminisolvens*, *Odoribacter* sp. DSM 112344, *Paraprevotella* sp., and *Parabacteroides* sp. AF19_14. *Lachnospira*, which is generally abundant in the mammalian gut, produces butyrate, an excellent nutrient for epithelial cells, while also exhibiting immunomodulatory and anti-inflammatory properties. A high abundance of *Lachnospira* is associated with intestinal health and confers resistance to intestinal inflammation [37,38]. *Schaedlerella arabinosiphila*, belonging to the family Lachnospiraceae, was anaerobically isolated from the feces of C57BL/6J mice using D-arabinose as the sole carbon source, demonstrating efficient utilization of both D-arabinose and L-arabinose for growth [39]. In our study, *Schaedlerella arabinosiphila* was identified as a differential microorganism in the gut of plateau zokors from the high-altitude group. Its ability to efficiently utilize arabinose and degrade plant polysaccharides may assist the host in adapting to varying food resources and extreme plateau environments across altitudes, thereby playing a key role in energy metabolism and survival adaptation. *Muribaculum gordoncarteri*, a strictly anaerobic gut symbiont with significant polysaccharide-degrading capabilities, may compensate for food resource scarcity under high-altitude hypoxic conditions by enhancing host energy utilization. This positions it as a crucial microorganism for plateau zokors in adapting to cold and hypoxic extreme environments. *Odoribacter*, another anaerobic bacterium commonly found in the mammalian gut, plays a vital role in maintaining intestinal barrier integrity through the production of short-chain fatty acids, particularly butyrate [40]. *Paraprevotella* exhibits a high capacity for degrading complex plant-derived polysaccharides and is involved in host amino acid metabolism as well as the regulation of gut homeostasis [41]. It may enhance intestinal anti-inflammatory capacity, mitigate hypoxia-induced intestinal damage, and indirectly optimize the host’s nutrient absorption efficiency by modulating the gut microecological balance [41]. *Parabacteroides* serves as a core gut symbiont in animals, performing functions such as polysaccharide degradation, regulation of intestinal immunity, and modulation of glucose and lipid metabolism. It protects the intestinal barrier and suppresses inflammatory responses. *Parabacteroides* is recognized as a promising target for disease intervention; however, its current status and outstanding issues require further clarification [42]. This study indicates that the colonization of *Parabacteroides* in plateau zokors suggests this genus can adapt to various altitudinal environments, aiding the host in utilizing plant-based foods and maintaining gut homeostasis.

Collectively, similar to other high-altitude mammals, plateau zokors adapt to extreme environments, such as high-altitude hypoxia, by altering the composition and abundance of their gut microbiota, thereby facilitating physiological adaptation.

With increasing altitude, the metabolites of plateau zokor—including bile acids and amino acids—undergo significant adaptive remodeling. Bile acids play critical roles in glucose and lipid metabolism, energy homeostasis, and immune regulation [12,43]. Consistent with our previous findings [44], the proportion of non-12-hydroxy bile acids increased, while that of 12-hydroxy bile acids decreased in high-altitude plateau zokor. Furthermore, our results revealed that as altitude increases, serum levels of UDCA and TUDCA significantly rise, while DCA significantly declines. These changes in bile acid composition may represent an adaptive metabolic strategy for extreme high-altitude environments. Additionally, gut microbiota regulate host amino acid catabolism and anabolism, thereby influencing energy metabolism, lipid metabolism, and oxidative stress [44]. Integrating metagenomic and metabolomic analyses, we identified that the differential microbes Candidatus *Amulumruptor caecigallinarius*, *Muribaculum gordoncarteri*, *Paraprevotella* sp., *Parabacteroides* sp., and *Heminiphilus faecis* exhibited significant positive correlations with 2-phenylpropionate and hydrocinnamic acid, while showing significant negative correlations with picolinic acid, aminocaproic acid, and suberic acid. Similarly, *Bacteroides graminisolvens* and *Staphylococcus aureus* demonstrated analogous correlation patterns; they were significantly positively correlated with DCA, methylcysteine, phenylalanine, and suberic acid, and significantly negatively correlated with UDCA and carnitine. These findings suggest that during high-altitude adaptation, plateau zokor selectively enrich specific functional microbial groups to coordinately regulate bile acid, energy, amino acid, and fatty acid metabolism. This represents a key regulatory mechanism that enables plateau zokor to adapt to extreme environments, including hypoxia and cold.

In plateau zokors, high-altitude adaptation is associated with a significant up-regulation of gut microbial pathways involved in nucleotide, thiamine, oxidative phosphorylation, and riboflavin metabolism, alongside a notable down-regulation of cysteine and methionine metabolism pathways. Nucleotides, as the body’s primary energy currency, play a crucial role in host metabolic and physiological regulation, including immune modulation [45]. Thiamine is phosphorylated to its active coenzyme form, thiamine pyrophosphate, which is essential for key metabolic reactions [46]. The up-regulation of nucleotide and thiamine metabolism implies an elevated overall metabolic rate in high-altitude individuals, likely sustaining higher energy metabolism under hypoxic conditions. Carbohydrate catabolism primarily occurs through glycolysis and oxidative phosphorylation, generating energy for physiological functions [47]. The marked up-regulation of oxidative phosphorylation further indicates enhanced energy acquisition in high-altitude plateau zokors. Riboflavin inhibits cholesterol biosynthesis, aiding in the maintenance of hepatic lipid transport, prevention of lipid peroxidation and accumulation, and regulation of lipid metabolism [48]. Consequently, the observed up-regulation of riboflavin metabolism suggests a role in lipid metabolism modulation, potentially as part of the adaptive response to high altitude.

In summary, plateau zokors exhibit significant altitude-dependent adaptations in gut microbiota composition, functionality, and serum metabolite profiles. These differential microbes and metabolites exhibit specific associations, and their coordinated regulation of key physiological processes in the host facilitates adaptation to high-altitude stressors, primarily hypoxia and low temperatures. This study offers novel insights into the mechanisms by which plateau mammals adapt to extreme environments.

## Data Availability

The raw metabolomics and metagenomic sequencing data generated in this study will be deposited in the National Genomics Data Center (NGDC, China National Center for Bioinformation) upon acceptance of the manuscript. Corresponding BioProject accession numbers will be included in the revised version.
